# Exploring nurse perceptions and experiences of resilience: a meta-synthesis study

**DOI:** 10.1186/s12912-021-00803-z

**Published:** 2022-01-19

**Authors:** Eun Young KIM, Sung Ok CHANG

**Affiliations:** 1 College of Nursing, Korea University, Seoul, Republic of Korea; 2 College of Nursing, and BK21 FOUR R&E Center for Learning Health Systems, Korea University, Seoul, Republic of Korea

**Keywords:** Resilience, Nurses, Perception, Experiences, Qualitative review

## Abstract

**Aim:**

To understand nurse resilience by integrating the qualitative research results on nurses’ resilience-related experiences.

**Methods:**

We applied the seven steps of the meta-ethnographic process by Noblit and Hare (1988). Five databases (PubMed, EMBASE, Web of Science, CINAHL and PsycINFO) were used to search for relevant studies published from January 2011 to September 2021.

**Results:**

Sixteen qualitative studies were included. The four themes of “self-development based on one’s inner self”, “fostering a positive attitude towards life”, “developing personal strategies for overcoming adversity” and “building professionalism to become a better nurse” illustrate that they want to improve their inner strength and develop themselves through self-examination.

**Conclusion:**

In this study, we examined nurse resilience, and the results can provide fundamental conclusions useful for the development of an intervention study to improve nurse resilience.

**Supplementary Information:**

The online version contains supplementary material available at 10.1186/s12912-021-00803-z.

## Introduction

Nurses are the largest occupational group in the medical health field. They account for approximately 59% of the total global medical health workforce [1], and play a very important role in patient care. Globally, in recent years the nursing profession has been a rapidly growing sector of the workforce [2]. However, nurses suffer stress and burnout related to their job, and this has a very negative effect on their mental health [3]. Problems that negatively affect the mental health of nurses can have serious consequences, such as decreased nursing professionalism, poor quality of care, increased social and financial losses, and increased turnover and resignation [4, 5].

Nurse job satisfaction has been in decline over recent years, and nurses have been found to develop negative perceptions about their job [2]. Nurse job satisfaction is related to the turnover rate, which affects the quality of patient nursing care. As concern over nurse job satisfaction and burnout is increasing worldwide [6], timely studies of concepts that can ameliorate the negative job perceptions of nurses caused by their negative experiences are required.

Resilience is a positive concept that allows nurses to overcome stressful situations [7] and to adapt positively, resulting in the maintenance of their psychological well-being and mental health [8, 9]. In a recent study, nurse resilience was found to significantly reduced nurse burnout [10, 11]. Resilience is emerging as an important concept for reducing the psychological burden of nurses and increasing their physical and mental health, since resilience has been shown to have a mediating effect on the relationship between burn out and physical/mental health [12]. Recently an understanding of an individual’s culture is considered very important for understanding resilience [13], so when exploring resilience in nurses, a key occupational group in society, we need to pay attention to their culture.

## Background

Most people have life-threatening experiences or are exposed to one or more stressors in their lifetime [14]. Therefore, it is very important to improve and adapt mental health to aid recovery from the challenges and adversities one faces and to adapt positively. Since nurses in particular are exposed to wide-ranging stresses, the ability to overcome such adversity is particularly important for them. The concept of resilience originated in psychology [15, 16] and can be described as an individual’s characteristics, processes, and outcomes [15].

According to Ungar [17], studying resilience requires a contextualized approach because the dynamic partnerships between individuals and social ecosystems can lead to positive adaptation when individuals face difficulties. Given this perspective, the issue of culture is very important, and exploring resilience in the context of the culture to which nurses belong can help to properly identify the dynamic property of resilience nurses. An understanding of resilience can be effectively applied to improving the mental well-being of nurses.

Research into nursing resilience has been steadily improving, and over the last 10 years the importance of the concept of resilience has become emphasized as related research has rapidly increased. In particular, resilience has been proposed as a solution to burn out [10, 11, 18] and mental health issues [19, 20], which nurses frequently suffer from, and related research has been actively conducted.

Nurse resilience contains a complex and dynamic process that changes over time and according to the situation, embodying not only personal attributes but also external resources, and describes a nurse’s ability to adapt positively to stress and adversity [21].

Academic interest in nurse resilience has been increasing recently, and many qualitative studies are being conducted to explore the essence of nurse resilience. However, the diversity of these qualitative studies and the differences in their findings has hindered understanding of the core concepts of nurse resilience.

Qualitative meta-synthesis is a methodology for synthesizing and analyzing individual qualitative research [22]. This methodology is recognized as a useful tool for analyzing the meaning, experience, and perspectives that participants’ express [22]. It can help accumulate knowledge and derive expanded knowledge and new interpretations from the the areas of research and phenomena suggested by the results of existing studies [23]. The methodology also enables more specific suggestions for future studies [22].

Noblit and Hare developed one of meta-synthesis method, meta-ethnography in 1988 [24]. This method has the potential to lower study duplication, create new research questions, and promote higher-level analyses [25].

In this study, the results of qualitative studies on nurse resilience are integrated, a new interpretations are attempted. This study will help provide fundamental information for the research and development of interventions to improve nurse resilience. The purpose of this study was to systematically review and synthesize the qualitative evidence on the nurse resilience experience.

### Aim

The aim of this study is to understand nurse resilience and to suggest directions for future research through the process of synthesizing and integrating qualitative research results on nurse resilience-related experiences.

## Methods

### Design

We used a meta-synthesis methodology, which provides broad understandings of social phenomena, to integrate the findings of qualitative studies [26]. Since meta-ethnography is an interpretive approach suitable for higher-level analyses and the formation of new interpretations beyond the discoveries of individual qualitative research [24], it is suitable for the purpose of this study, which is to synthesize and newly interpret research on nurse resilience experience. We followed the meta-ethnography method, which is suitable for both preserving the interpretations of the primary data and forming new interpretations, theories and models [27]. We applied the seven steps of the meta-ethnographic process by Noblit and Hare [24]: (a) getting started, (b) deciding what is relevant to the initial interest, (c) reading the studies, (d) determining how the studies are related, (e) translating the studies into one another, (f) synthesizing those translations, and (g) expressing the synthesis.

The research questions were:

1. “How do nurses overcome adversity?”
2. “What are the characteristics of nurses resilience experiences?”

This review was prepared in accordance with ENTREQ (Enhancing transparency in reporting the synthesis of qualitative research Statement) guidelines [28]. This meta-synthesis study was registered (CRD42021275787) with PROSPERO, which is the International Register of Systematic Reviews.

*Phase 1. Getting started & Phase 2. Deciding what is relevant to the initial interest.*

### Search methods

We used the *narrative* literature review method. As this method is mainly used for searching literature representing the entirety of the phenomenon of interest, it is suitable for meta-synthesis studies [25].

**Fig. 1:**
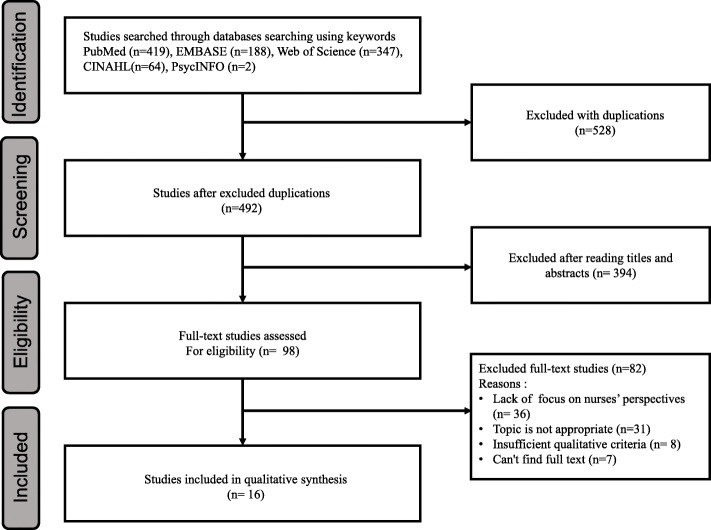
Flowchart of systematic review for literature selection

Before the literature search, the two authors discussed the search strategy and databases to be used to search for appropriate articles that meet the purpose and inclusion criteria of this study. The authors selected PubMed and EMBASE, which are considered the most important databases for literature searches in the medical field [29], and included Web of Science to broaden the search to the field of social science. In addition, CINHAL, a nursing database, and PsycINFO were important to include due to the psychological nature of the concept of resilience. Thus, literature searches were carried out across five databases overall (PubMed, CINAHL, EMBASE, Web of Science and PsycINFO). The representative search terms used in the search were “Nurses”, “Resilience, Psychological”, “Qualitative Research”, and “Hermeneutics” from the list of Medical Subject Headings (Mesh terms). The search terms were adapted according to the index terms of each database. The search terms were used with the Boolean operators “AND” and “OR” in different combinations. The search strategy of this study is presented in supplementary material Table S1. A flow chart of the systematic review of literature selection process of the present research is presented in Fig. 1. A review study on a similar subject was conducted in 2012 [30]. In our search process we found that relevant studies had increased rapidly since 2011. Therefore, we limited the search results to the last 10 years to achieve a synthesis focusing on the latest research results.

The inclusion criteria required qualitative studies that were: (a) aimed at exploring the resilience of nurses, (b) published from January 2011 to September 2021 (c) peer-reviewed journals (d) published in English, and (e) full-text searchable. The exclusion criteria were (1) nurse experiences were not reported separately, (2) mixed methods were used but qualitative data could not be extracted or (3) necessary qualitative depth was lacking in the data analysis.

Through the search procedure, 1020 studies were identified. 528 studies were excluded as duplicates in Endnote, and the two authors independently reviewed each title and abstract, thereby excluding 394 studies. When the two authors independently reviewed the full-texts and discussed any disagreements, 82 studies were excluded because of a lack of focus on nurse perspectives, an inappropriate topic, insufficient qualitative criteria, or because the full text could not be found. After this process, 16 studies remained for inclusion (Fig. 1).

### Quality appraisal

The Critical Appraisal Skills Programme (CASP) checklist, which contains 10 questions for assessing the reliability and rigor of individual studies, was used to appraise the 16 studies [31]. Two authors independently evaluated the 16 included studies using the CASP checklist. After the appraisal, the two authors compared the CASP results, and any disagreements were resolved through discussion. The degree to which the studies met CASP was evaluated to be 70% for 4 studies, 80% for 9 studies, and 90% for 3 studies. Since all the studies were evaluated as being 70% or higher, none were excluded from the evaluation process (Table 1).

**Table 1:** Summary of the included studies

Article. No.	Author, year/Country	Research type	Aims	Sample size (F:M)	Age of participants (in years)	Nursing experience (years)	Working department	Data collection	Data analysis	Percentage that meets CASP
A1	Mealer et al., 2012/USA	Qualitative study	To identify mechanisms employed by highly resilient ICU nurses to develop preventative therapies to obviate the development of PTSD in ICU nurses	27, (27:0)	mean: 46	Total: mean18.5	Intensive Care Unit	Semi-structured telephone interviews	Thematic analysis	80%
A2	Shimoinaba et al., 2015/Japan	Qualitative study	To explore the nature of nurses’ resilience and the way it is developed	18, (18:0)	29–53 mean: 37.8	Total: 7–26 In this department: 2–8, mean37.8	Palliative Care Unit	Face to face in-depth interviews	Grounded theory	80%
A3	Cope et al., 2016/Australia	Qualitative portraiture methodology	To explore residential aged care nurses working in interim, rehabilitation and residential aged care perceptions of resilience	3,(not reported),	32–57	Total: mean28	An aged care environment	Semi-structured interviews painting with words	Thematic analysis	70%
A4	Cope et al., 2016 (2) /Australia	Qualitative portraiture methodology	To explore why nurses chose to remain in the Western Australian workforce; to develop insights into the role of resilience of nurses to manage the context of nursing work; and, to identify the key characteristics of resilience displayed by those nurses	9,(not reported)	Not reported	Not reported	Interim and residential aged care, academic setting, tertiary acute care setting	Individual interviews, field notes, memos and gesture drawings interviews	Phenomenology	70%
A5	Tubbert, 2016/USA	Qualitative study	To explore the resiliency characteristics of certified emergency nurses	16 (68.8%:31.2%)	Mean: 50	Total: 30 In this department: 20	Emergency department	Face to face interview	Content analysis	80%
A6	Benade et al., 2017/South Africa	Explorative descriptive qualitative research	To explore and describe the strengths and coping abilities of nurses caring for older persons and to formulate recommendations to strengthen their resilience	43 (43:0)	Not reported	Total: not reported In this department: < 6 month: 2 1 year < 5 years: 5 5 years < 10 years: 4 > 10 years: 27	Aged care department in an urban environment	Focus group interview	Content analysis	70%
A7	Marie et al., 2017/UK	Interpretive qualitative design	To observe and describe the environment within community mental health workplaces, to explore the challenges facing Palestinian community mental health nurses (CMHNs) inside and outside their workplaces, and to examine their sources of resilience	15 (8:7)	24–60	Not reported	Mental health workplace	Face-to-face in depth interviews	Thematic analysis	80%
A8	Prosser et al., 2017/Canada	Interpretative phenomenological method	To understand how registered nurses in the acute psychiatric setting develop resilience to sustain his or her practice.	4(not reported)	Not reported	Total: 2–21 In this department: 2–16	Acute psychiatric units	Semi-structured face-to- face interview	Interpretative phenomenological analysis.	90%
A9	Wahab et al., 2017/Singapore	descriptive qualitative design using Photovoice	To explore the new graduate nurses’ accounts of resilience and the facilitating and impeding factors in building their resilience	9 (6:3)	Mean: 24	Total: mean 1 In this department: not reported	Oncology, General Medicine, General Surgery, Psychiatry and Paediatric wards	Focus group interview, photographs	Content analysis	80%
A10	Imani et al., 2018/Iran	Phenomenology study	To explore Iranian hospital nurses’ lived experiences of intelligent resilience	10 (4:6)	34–52	Total: 11–28 In this department: not reported	Different types of wards	In-depth interview	The Colaizzi’s (1978) seven-step approach	70%
A11	Jackson et al., 2018/UK	Grounded theory	To better understand nurse burnout and resilience in response to workplace adversity in critical care	11 (11:0),	20s:5, 30s:3, 40s:1, 50s:2	Total: 4–36 In this department: not reported	Intensive care unit	Open-ended interviews	Grounded theory	90%
A12	Ramalisa et al., 2018/South Africa	Empirical qualitative research	To explore and describe how to strengthen the resilience of nurses in a work environment with involuntary mental health care users.	24(not reported)	Not reported	Total: 2–8 In this department: not reported	Psychiatric ward	Open-ended interview	Thematic analysis	80%
A13	Ang et al., 2019/Singapore	Qualitative grounded theory design	To generate a comprehensive account of the experiences of nurses as they cope with stress and demands of work, and to develop knowledge of the phenomenon of resilience among nurses.	15 (15:3),	24–68 mean:38	Not reported	General hospital	Individual interviews	Glaserian constant comparison method	80%
A14	Ang et al., 2019 (2)/Singapore	Photovoice study	To explore the meaning of resilience to nurses and their perceived resilience enhancing factors	8 (7:1)	27–68	Not reported	Accident and emergency department	Focus group interview, photo	Content analysis	80%
A15	Lin et al., 2019/Taiwan	Construction-grounded theory	To explore and understand the experiences of resilience among nurses in an overcrowded emergency department (ED)	13 (13:0)	23–39	Total: not reported In this department: 2–17	Emergency department	In-depth interview	Construction-grounded theory	90%
A16	Udod et al., 2021/Canada	Qualitative study	To investigate the role stressors, and how coping strategies cultivated nurse managers’ resilience in rural workplaces.	16 (15:1)	30s: 5 40s:9 Over 60:2	Total: mean4.6, 10–35 In this department: mean 7.28, 1–17	Rural site in western Canada	Individual semi-structured interview	Thematic analysis	80%

F:female; M:male; CASP: Critical Appraisal Skills Programme checklist

*Phase 3. Reading the studies & Phase 4. Determining how the studies are put together.*

### Data extraction

Two authors independently reviewed the studies in detail. Each author read the studies line by line and tried to derive meaningful concepts by extracting codes. Data extraction for the study was performed using a custom form in Microsoft Excel, including author details, participant characteristics, methods, and original citations [27]. Disagreements between the authors were solved through discussion.

*Phase 5. Translating the studies into one another, Phase 6. Synthesizing the translations & Phase 7. Expressing the synthesis.*

### Data synthesis

Based on the meta-ethnography process [24], the 16 studies were independently read repeatedly by the two authors, data analysis was performed, and then the extracted data were summarized as concepts. In more detail, after organizing the papers in chronological order, the two authors independently read the 16 studies, repeatedly. They then summarized and extracted meaningful concepts and themes. The two authors compared the themes and concepts of the first paper with those of the second paper, and then compared the common themes of those two papers with the third paper to derive concepts and themes. To synthesize the key concepts and broader themes, this process was repeated until the final study. To extract the key concepts, the studies were read several times, and the key concepts of each study were listed and analyzed for comparison. The key concepts were formed based on the ‘first-order construct’ of the study. In the meta-ethnography analysis process, the data is divided into ‘first-order construct’, ‘second-order construct’, and ‘third-order construct’ [32]. The ‘first-order construct’ is the original study participant’s daily language as expressed in their own language in the original study, the ‘second-order construct’ is the researcher’s interpretation based on the ‘first-order construct’, and ‘third-order construct’ is a new interpretation of ‘second-order construct’ [32, 33]. In this study the authors of the current paper extracted the key concepts of the ‘first-order construct’, then compared the similarities and differences to form the ‘second-order construct’, and finally derived the ‘third-order construct’, which represented the main themes of the current study, by abstracting the ‘second-order construct’. During this process of analysis and synthesis, the two authors continued to discuss their differences of opinion based on their respective academic and clinical backgrounds. The final analysis step, “expressing the synthesis,” was accomplished by collating the discussion results. In order to confirm the value of the data, the researchers asked one incumbent nurse and one nursing professor to confirm the appropriateness of the expression of the results and the choice of terminology, and after the discussion the results were amended accordingly and finalized. The quotes that best expressed each sub-theme were identified and are presented in the results.

### Ethical consideration

As this study is a review study, as a meta-synthesis, human participants were not included. Therefore, an ethical committee review was not required.

## Results

Sixteen qualitative studies were included in this review study and the publication years of the included studies were from 2012 to 2021. A total of 241 nurses participated in the included reviewed studies. Their ages varied widely, from those in their 20s to those in their 60s, and they were generally of female gender. Their working departments were varied, and included the ICU, the geriatric ward, the emergency room, the psychiatric ward, and the general ward. The studies were conducted in Africa, Australia, Canada, Iran, Japan, Singapore, Taiwan, USA, and UK. In other words the studies were conducted in various countries around the world (Table 1). This study yielded four comprehensive themes of nurse resilience experience. These were ‘self-development based on one’s inner self’, ‘fostering a positive attitude towards life, ‘developing personal strategies for overcoming adversity’, ‘building professionalism to become a better nurse’. Table 2 shows the key concepts from the first-order constructs, the second order constructs, and the synthesized themes.

**Table 2:** Synthesized themes of nurses’ resilience experience

Key concepts from first-order constructs	Sub-themes	Synthesized themes
Self-reflection ^A2, A3, A4, A5, A8, A11, A13, A14, A16^ Focusing on the present ^A2, A5, A8, A9, A11, A12, A13, A14^ Accept the situation not avoiding ^A8, A9, A11, A12, A13, A14^ Believe in one’s ability ^A2, A5, A6, A9, A10, A12, A13, A14^ Expressing their feelings honestly ^A2, A6, A10, A11, A13^ Recognizing the warning signs of stress ^A5, A9, A11, A13^ Pay attention to physical and emotional demands ^A5, A6, A8, A10, A11, A16^	1. Recognizing and acknowledging the signs of adversity	I. Self-development *based* on one’s inner self
Trying to maintain physical health ^A3, A4, A11^ Perceiving self-efficacy ^A5, A6, A9, A11, A13, A14, A15^ Developing various ways of self-care ^A2, A3, A4, A9, A11, A13, A14, A15^ Having self-confidence ^A5, A6, A9, A11, A13, A14, A15^ Trying to control emotions ^A2, A5, A6, A9, A11, A13, A14, A15^ Learning from others’ experience and expertise ^A2, A3, A4, A6, A7, A9, A10, A16^ The taking on new challenge ^A2, A3, A4, A5, A9, A11, A13^ Flexible thinking to solve an issue ^A2, A5, A9, A11, A12, A13, A14^ Preserving and moving forward ^A5, A9, A11, A13, A14^	2. Striving to grow themselves	
Keeping optimistic view in life ^A1, A3, A4, A5, A6, A7, A9, A14, A15^ Maintaining a sense of humor at work ^A3, A4, A6, A11^ Gratitude for life ^A3, A4, A6, A7, A12^ Living a life of helping others ^A2, A3, A4, A6, A15^ Remembering good experiences from the past ^A1, A2, A5, A6, A15^ Finding joy and pride in what they do ^A1, A5, A6, A11, A15^	3. Accepting life positively	II. Fostering a positive attitude towards life
Living a regular and healthy life ^A1, A2, A5, A11^ Enjoying a variety of leisure activities ^A1, A2, A5, A11, A13^ Creating their own hobbies ^A1, A2, A5, A13^ Developing their own personal coping behaviors ^A1, A2, A5, A11, A13^	4. Enjoying their *own* life	
Avoiding stress ^A1, A9, A10, A11^ Keeping work-life balance ^A5, A6, A8, A10, A11, A12^ Keep distance life from work (Setting boundaries between home and work) ^A5, A6, A8, A10, A11, A12^	5. Staying away from stress	III. Developing personal strategies for overcoming adversity
Comfort from friendships ^A1, A3, A4, A5, A7, A10, A11, A12, A14, A15, A16^ Getting help from family relationships ^A1, A3, A4, A5, A7, A11, A12, A14, A15, A16^ Maintaining good relationship with colleagues ^A1, A2, A3, A4, A5, A7, A10, A11, A12, A13, A14, A15, A16^ Positive role model ^A1, A2, A6, A11, A12, A15^ Sharing their feelings with someone whom they trust ^A1, A2, A5, A6, A11, A12, A13, A14, A15, A16^ Get help from experts ^A6, A9, A12^	6. Getting comfort through positive interpersonal relationships	
Developing self for the future ^A2, A3, A4, A6, A7, A9, A13, A16^ Preparing for new assignments ^A2, A3, A4, A6, A7, A9, A14, A16^ Adaptation to a new situation ^A2, A7, A9, A13, A14, A16^ Prioritizing work ^A5, A8, A13, A14, A15, A16^	7. Planning their life for a better future	IV. Building professionalism to become a better nurse
Sense of professional pride ^A2, A3, A4, A6, A9, A10, A11, A12, A13, A15, A16^ Value of being a nurse ^A2, A3, A4, A6, A9, A11, A12, A13, A15, A16^ Satisfaction with career ^A2, A3, A4, A6, A9, A11, A13, A14, A15, A16^ Passion to work ^A2, A3, A4, A6, A9, A10, A11, A12, A13, A15, A16^ Developing their knowledge and skills ^A2, A3, A4, A6, A9, A12, A13, A15^	8. Building self-esteem by thinking about the value of a job	

### Theme I. Self-development based on one’s inner self

The sub-themes included in theme 1 were “recognizing and acknowledging signs of adversity” and “striving to develop oneself”. The nurses tried to find solutions by focusing on the signals that they had encountered adversity, and making an effort to grow through that adversity rather than collapse under it.

### Sub-theme 1. Recognizing and acknowledging the signs of adversity

The nurses did not deny the signs of adversity, but recognized and acknowledged them. The nurses looked inside themselves to find problems and tried to reflect on themselves *[A2-A5,A8,A11,A13–14,A16]*. They tried to focus on the current situation *[A2,A5,A8–9,A11–14]* and showed an attitude of acceptance of the situation they were in rather than one of avoidance *[A8–9,A11–14]*. They believed in their abilities *[A2,A5,A6,A9–10,A12–14]*, tried to express their feelings honestly *[A2,A6,A10,A11,A13]*, and tried to recognize the warning signs of stress rather than ignore them *[A5,A9,A11,A13]*. They showed themselves as focusing on the state and demands of their bodies and emotions *[A5–6, A8,A10–11,A16]*.

*“It is difficult....I feel it is a most difficult thing to reflect on my own feelings. I can understand other people, but I do not understand myself. I think I have experienced a kind of burnout.”* (A2).

*“I thought of ways when something happens so that one can go away, sit down and reflect and then maybe come up with whatever your own strategies are to come back stronger. So if the same thing comes at you again, you know where to run, which direction is faster”* (A11).

### Sub-theme 2. Striving to develop oneself

Rather than despair, the nurses wanted to grow on their own. They tried to maintain their physical health *[A3–4,A11]* and recognized a sense of self-efficacy *[A5–6,A9,A11,A13–15]*. In addition, they thought about and developed various ways to take care of themselves *[A2-A4,A9,A11–15].* They were confident that they could overcome the adversity *[A5–6, A9, A11, A13–15]* and tried to control their emotions *[A2,A5–6,A9,A11,A13–15].* Nurses tried not to ignore what others had to say and to learn from their experiences and expertise *[A2–4,A6–7,A9–10, A16]*. They tried new challenges *[A2–5,A9,A11,A13]* and had varied thoughts to solve problems *[A2, A5, A9, A11–14].* The nurses showed patience and a willingness to move forward *[A5,A9,A11,A13–14]*.

*“You know more or less that you have to do it and it is going to be tough going. The task could be from manageable to unmanageable depending on what is happening at the moment. I’m able to cope with stress.”*(A13).

### Theme II. Fostering a positive attitude towards life

In theme 2, the sub-themes included “accepting life positively” and “enjoying *their own* life”. In this theme, the power of nurse positivity is evident. The nurses showed that they were trying to overcome adversity with positivity. A positive view toward life and the desire to live their own lives gave the nurses strength in their professional lives.

### Sub-theme 3. Accepting life positively

The nurses maintained a optimistic view toward life when overcoming life adversities *[A1,A3–7,A9,A14–15]* and maintained a sense of humor at work *[A3–4,A6,A11]*. They were grateful for life *[A3–4,A6–7,A12]* and tried to heal themselves by living a life of helping others *[A2–4,A6,A15]*. They remembered the good memories they had in the past, and drew positive thoughts from them *[A1–2,A5–6,A15]*. They found enjoyment and pride in what they did *[A1,A5–6,A11,A15].*

*“But I try to look at the positive stuff*. *.. what we are able to do, what changes we were able to make as a result of a catastrophe or just a bad outcome. .. just pull my sleeves up and get in there and get it done and when I can, I do try to encourage communication and good feelings.”* (A5).

*““I think every type of nurse has their own type of black humor but I realized a lot of it is a coping mechanism and a way of protection to get yourself through the day”* (A11).

### Sub-theme 4. Enjoying their ownlife

Nurses tried to overcome the adversities they faced while enjoying life on their own terms *[A1–2,A5,A11]*. They tried to live a regular and healthy life, enjoy various leisure activities *[A1–2,A5,A11,A13]* and enrich their lives happily while creating their own hobbies *[A1–2,A5,A13].* They coped with the situation by developing their own coping behaviors for managing crisis situations *[A1–2,A5,A11,A13].*

*“You have to enjoy what you are doing. If you hate your work, it will be a constant stress. I feel that I don’t get stressed about it because I feel that no problem is difficult.”* (A13).

*“The joy of working is like a hurdle … like jumping over the hurdle. Each time I jump over a hurdle that I cross, there is always some satisfaction in the job.”* (A13).

### **Theme III.** Developing personal strategies for overcoming adversity

The sub-themes “staying away from stress” and “getting comfort through positive interpersonal relationships” were included in theme 3. Nurses were shown to develop their own strategies when overcoming adversities. They tried to stay away from situations that were stressful to them and formed their own defenses through positive interactions provided by wide-ranging human relationships. These methods of overcoming adversity were their own individual strategies learned through their own experiences.

### Sub-theme 5. Staying away from stress

Nurses had to face varied sources of stress, but tried to avoid them*,* such as by trying to not create stressful situations or focusing on stress *[A1,A9,A10–11].* They tried to maintain a work-life balance so that neither aspect became too large or too small, breaking the balance *[A5–6,A8,A10–12].* In addition, by thoroughly separating their work and life, they tried to thoroughly protect their private life, avoiding the intrusion of work. They tried to respect their own privacy *[A5–6,A8,A10–12].*

*“Sometimes, I can’t control myself. In such situations, I attempt to distance myself from that situation or the immediate environment. In these conditions, I ask my colleagues to continue care delivery and then, I leave the situation. I never stay in such a situation because I know that my presence will aggravate the problem. Thus, I leave that situation and start providing care to another patient.” *(A10).

“*I think that the only way for me to stay resilient is to keep stepping away from the bedside, because that’s where all the stress is for me, it’s at the bedside. You need to remove yourself from the situation*” “*I have to have this proper balance and this little routine to maintain a healthy, functional life, and I think the younger ones know that which is good.”* (A11).

### Sub-theme 6. Getting comfort through positive interpersonal relationships 

In their professional positions the nurses developed a variety of interpersonal relationships, including relationships with patients, colleagues, and families. Nurses received comfort from their relationships with friends *[A1,A3–5,A7,A10–12,A14–16]* and comfort and support from their families *[A1,A3–5,A7,A11–12,A14–16]*. The nurses tried to maintain good relationships with their colleagues and other nurses and as colleagues they helped each other in difficult situations *[A1–5,A7,A10–16]*. In addition, they tried to grow one step at a time by orientating their own future direction through role models and mentors they could imitate [A1–2,A6,A11–12, A15], and they shared their feelings with people they could trust [A1–2, A5–6, A11–16]. They also received help from a mental health professionals for stress management *[A6,A9,A12].*

*“It’s the people you work with. I have a lot of caring friends. I talk to my husband - he always backs me up. I think that how you deal with it..*. *with another manager’s support. We meet for lunch sometimes — a laughs the best way — we quite often see the funny side.”* (A4).

*“Talking to colleagues because they know the scope of your job. They know what is happening in your ward, so they will be able to understand better.”, “I have good friends to whom I can confide my problems. I think it’s important you don’t bottle up your feelings too much, because you know you can just self-destruct if you’re not able to handle it. They may not be able to solve the problem; a listening ear does help.”* (A13).

*“I’m very fortunate as I have a large network of friends and colleagues that I can safely vent to or discuss things with or bounce ideas off that aren’t my staff. And I found that you really need that. It is pretty much a lifeline whether you’re a front-line manager or if you’re a director you need to have that core group of people that you can call and say, ‘Am I crazy’”* (A11).

### Theme IV. Building professionalism to become a better nurse

In theme 4, the sub-themes “planning their life for a better future” and “building self-esteem by thinking about the value of a job” were included. The nurses planned their future to live a better life than just leaving their lives to adversity. They felt that they had to develop themselves for a better future, prepare for a new life, and adapt to a new situation. They thought they had to rearrange their work by considering their priorities. *This process led them to build their professionalism.*

### Sub-theme 7. Planning their life for a better future

In difficult situations, nurses thought about how to live their future. Even in difficult situations, the habit of planning for the future and thinking about the future rather than staying in the present and despairing was exhibited by nurses *[A2–4,A6–7,A9,A13,A16]*. They wanted to develop themselves, to prepare for new assignments including difficult situations [*A2–4,A6–7, A9,A14,A16]*, and to adapt well to new situations *[A2,A7,A9,A13–14,A16].* They re-prioritized their work and tried to work according to their priorities *[A5,A8,A13–16].*

*“I always take an experience as an opportunity to learn from it. To grow. I mean, no experience is bad. It may be a bad experience but you can learn from it and try to move on and try to make things better”* (A13).

### Sub-theme 8. Building self-esteem by thinking about the value of a job

In difficult situations, nurses tried to gain the strength to overcome the crisis by reflecting on themselves being nurses *[A2–4,A6,A9–13,A15–16]* and on the value of their job *[A2–4,A6,A9,A11–13,A15–16].* They took pride in their job, thought about the meaning and value of being a nurse, and tried to overcome crises while gaining satisfaction from the value of their work *[A2–4,A6,A9,A11,A13–16].* They had a passion for their work *[A2–4,A6,A9–16]* and wanted to develop their knowledge and skills as nurses and upgrade themselves through individual development *[A24,A6,A9,A12–13,A15].*

*“We are the backbone when patients come in. The nurse is the protector of the patient." *(A14) "*The patient survived because the nurse stuck out her hand and stopped the bleeding. I am proud of her.”* (A11).

## Discussion

As nurses play a very important role in the medical field, they are exposed to wide-ranging difficulties because of the high intensity of their work. Research on the resilience of nurses, a positive force to overcome this, has recently attracted attention in both research and practice. This study synthesized the result of qualitative research on nurse resilience to explore nurse experiences of overcoming adversity.

According to the result of this study, when nurses felt that they were going through a difficult situation, they tried to recognize and acknowledge that situation by exploring their inner selves. They focused on themselves and tried to find out exactly what their situation and problems were through self-exploration and not evading issues. This can be thought of as a preparation process for problem solving. In the context of the more general resilience attributes found by previous studies, it can be noted that the nurse attributes discovered by this study, such as a belief in self-efficacy and a desire to improve oneself, are aspects of resilience in general [34]. In addition, the results of this study can support the results of previous studies that internal protection factors such as self-efficacy, optimism, emotional intelligence and self-management should be covered in the training of resilient nurses [35]*.*

However, nurse resilience included striving for self-development while focusing on reality. These results show that nurses in a crisis situation have a strong tendency to rapidly grasp problems, trust themselves and solve problems quickly. Also, the nurses showed that they wanted to solve problems and grow step by step rather than remaining in crisis. The subjects of this study were incumbent nurses in their 20s to 60s, including relatively young subjects, and it likely that the specific nature of the active and developmental results derived in this study reflect all of them having the profession of nursing in common.

Nurses tried to overcome adversity by accepting life positively and enjoying their own lives. In previous studies, this positivity property as a component of resilience was found to be universal across varied subjects, as a part of the concept of general resilience [33, 36] and the resilience of chronic disease patients [37]. In addition, nurses showed the characteristics of wanting to be challenged in life and enjoy the life given to them. This can be seen as showing an active attitude to life, and, since nurses are generally made up of healthy young people, this showed a different aspect of resilience from those shown by patients who are highly dependent on their families and medical staff [37, 38]. A positive and active attitude towards life could be an important factor to focus on in the development of future intervention research aiming to improve nurse resilience.

Nurses exhibited the resilience characteristics of avoiding stress and separating their lives from stress when overcoming adversity. They showed that they were trying to overcome adversity by guaranteeing the quality of their own lives. This characteristic results from the professional stress of a nurse, reflects the substantial stress they face at work, and is something we should pay attention to. The results of this study can support the findings of previous studies that nurses recognized work-life separation as a very important factor when considering resilience [39]*.* Nurses got comfort from wide-ranging human relationships. The human relationships of nurses were characterized by wanting to develop their relationships between colleagues to an intimate level, particularly friendship, and them securing comfort from these relationships with such close colleagues. Considering these characteristics of nurse resilience shows the need when developing future intervention studies to improve nurse resilience to create resilience programs that consider peer relationships.

As the nurses overcame adversity, they try to adapt themselves by developing themselves and preparing for new situations. They try to organize their lives while arranging work priorities. Nurses tried to overcome adversity by planning for the future and developing one step at a time. In addition, in the face of adversity they took pride in being nurses by reflecting on why they chose their profession and reminding themselves of its value. By raising their job satisfaction and passion for their work, their self-esteem was also raised. They wanted to grow further as nurses by constantly exploring and developing job-related knowledge and skills, and tried to overcome the difficult situations they faced through the process of growth. The aspect of resilience, that contains the meaning of growth, has been revealed in previous studies [40]. However, the resilience of nurses has more specific meanings than the previous concept of resilience in that nurses plan for a better future, value the professional meaning of nursing, and want to develop as nurses. This characteristics of the resilience of nurses who plan for the future in difficult situations and want to develop themselves further can suggest a direction for intervention research to enhance nurse resilience.

Due to the COVID-19 pandemic of recent years, many medical staff, including nurses, are struggling in the medical field. Several studies have been published that show that, in some countries, facing this difficult situation has lowered the resilience of nurses [41, 42]. Resilience is the strength to overcome such crisis situations, and the importance of the ability of nurses to overcome these global medical crises is increasingly being emphasized [43]. Many studies have emphasized the need for intervention research to improve nurse resilience [44]. The results of this study can provide fundamental data on what factors to focus on when developing intervention studies to improve nurse resilience, which has been lowered in the pandemic situation. We need to focus on the inner self and plan interventions that will improve that aspect. Also, interventions that rebuild positive strength and allow nurses to overcome adversity and grow individually will be very helpful for them to overcome adversity. These varied approaches can be expected to give positive strength to nurses, particularly in the currently challenging medical field.

This study helps the understanding of resilience in nursing, and provides an appropriate lens for a contextualized approach to resilience research. This is very meaningful data from a socio-ecological point of view and emphasizes the importance of interactions between individuals and society [17], and is expected to ultimately play a positive role in the development of society.

This study has two strengths. First, the included studies are from very diverse countries, such as the United States, Japan, Australia, South Africa, the United Kingdom, Canada, Singapore, Iran, and Taiwan, reflecting studies of various cultures. Therefore, this study can provide universal knowledge that is applicable worldwide. Second, of the literature used in this study, all 16 articles were found to satisfy the quality evaluation criteria by 70% or more, so the study is based on an analysis of relatively high quality documents of well-organized structure.

Despite these strengths, this study has limitations. The nurses included in the literature were from wide-ranging sub-fields, and the specific stress characteristics of the particular departments were not reflected in the result. Therefore, in the future, conducting qualitative research on the resilience of nurses across nursing sub-fields is suggested. To resolve this, meta-synthesis studies could be conducted to elucidate the characteristics of nurses within more specific fields.

### Future research

Resilience enhancement is an approach that maximizes human inner strength, and we can suggest an intervention that maximizes inner human resources by using mobile resources featuring excellent individual accessibility. Mobile health intervention applications have developed rapidly lately and their usefulness is recognized in nursing [45]. Recently, mobile health (mHealth) using smartphones for psychotherapy has been attracting attention as useful for the upcoming post-corona era [46]. Mobile applications can be useful resources for problem solving, and providing real-time information and stress reduction strategies for nurses in a variety of healthcare fields and settings [47]. In addition, since previous studies found smartphone app-based resilience interventions effective for resilience, emotional regulation, and psychological health promotion, the development of a smartphone app-based nurse resilience enhancement program can be expected based on the results of this study [48]*.*

## Conclusion

Emphasizing resilience, a positive force for overcoming adversity, to nurses can play a very significant role in improving the quality of nursing care. In this study, nurses showed resilience to grow and develop themselves by focusing on their inner selves and finding ways to solve problems on their own. These can be considered as categories of nurse resilience and provide a framework to guide the development of an intervention program for improving nurse resilience. Based on the results of this study, to improve the quality of nursing care we should try to develop varied intervention programs that enhance nurses’ inner strength.

## Supplementary Information

**Additional file 1.**

## Data Availability

All data generated or analyzed during this study is included in this published article.

## Appendix 1

### List of Synthesized Studies

**A1.** Mealer M, Jones J, Moss M. A qualitative study of resilience and posttraumatic stress disorder in United States ICU nurses. Intensive Care Med. 2012;38(9):1445–51. 10.1007/s00134-012-2600-6

**A2.** Shimoinaba K, O’Connor M, Lee S, Kissane D. Nurses’ resilience and nurturance of the self. Int J Palliat Nurs. 2015;21(10):504–10. 10.12968/ijpn.2015.21.10.504

**A3.** Cope VC, Jones B, Hendricks J. Residential aged care nurses: portraits of resilience. Contemp Nurse. 2016;52(6):736–52. 10.1080/10376178.2016.1246950

**A4.** Cope V, Jones B, Hendricks J. Why nurses chose to remain in the workforce: Portraits of resilience. Collegian. 2016;23(1): 87–95. 10.1016/j.colegn.2014.12.001

**A5.** Tubbert SJ. Resiliency in emergency nurses. J Emerg Nurs. 2016;42(1):47–52. 10.1016/j.jen.2015.05.016

**A6.** Benade P, du Plessis E, Koen MP. Exploring resilience in nurses caring for older persons. Health SA. 2017;22:138–49. 10.1016/j.hsag.2017.01.003

**A7.** Marie J, Hannigan B, Jones A. Resilience of nurses who work in community mental health workplaces in Palestine. Int J Ment Health Nurs. 2017;26: 344–54. 10.1111/inm.12229

**A8.** Prosser SJ, Metzger M, Gulbransen K. Don’t just survive, thrive: understanding how acute psychiatric nurses develop resilience. Arch Psychiatr Nurs. 2017;31(2):171–6. 10.1016/j.apnu.2016.09.010

**A9.** Wahaba SNB, Mordiffi SZ, Ang E, Lopez V. Light at the end of the tunnel: New graduate nurses’ accounts of resilience: A qualitative study using Photovoice. Nurse Educ Today. 2017;52:43–9. 10.1016/j.nedt.2017.02.007

**A10.** Imani B, Kermanshahi SMK, Vanaki Z, Lili AK. Hospital nurses’ lived experiences of intelligent resilience: A phenomenological study. J Clin Nurs. 2018;27(9–10), 2031–40. 10.1111/jocn.14310

**A11.** Jackson J, Vandall-Walker V, Vanderspank-Wright B, Wishart P, Moore SL. Burnout and resilience in critical care nurses: A grounded theory of Managing Exposure. Intensive Crit Care Nurs. 2018;48:28–35. 10.1016/j.iccn.2018.07.002

**A12.** Ramalisa RJ, du Plessis E, Koen MP. Increasing coping and strengthening resilience in nurses providing mental health care: Empirical qualitative research. Health SA. 2018;23:1094. 10.4102/hsag.v23i0.1094

**A13.** Ang SY, Uthaman T, Ayre TC, Lim SH, Lopez V. Differing pathways to resiliency: A grounded theory study of enactment of resilience among acute care nurses. Nurs Health Sci. 2019;21:132–8. 10.1111/nhs.12573

**A14.** Ang SY, Uthaman T, Ayre TC, Lim SH, Lopez V. A Photovoice study on nurses’ perceptions and experience of resiliency. J Nurs Manag. 2019;27(2):414–22. 10.1111/jonm.12702

**A15.** Lin C-C, Liang H-F, Han C-Y, Chen L-C, Hsieh C-L. Professional resilience among nurses working in an overcrowded emergency department in Taiwan. Int Emerg Nurs. 2019;42: 44–50. 10.1016/j.ienj.2018.05.005

**A16.** Udod S, Care WD, Graham JM, Henriquez N, Ahmad N. From coping to building nurse manager resilience in rural workplaces in western Canada. J Nurs Manag. 2021;Online ahead of print. 10.1111/jonm.13350
