# Correction to: Exploring nurse perceptions and experiences of resilience: a meta-synthesis study

**DOI:** 10.1186/s12912-022-00819-z

**Published:** 2022-02-15

**Authors:** Eun Young Kim, Sung Ok Chang

**Affiliations:** 1 College of Nursing, Korea University, Seoul, Republic of Korea; 2 College of Nursing, and BK21 FOUR R&E Center for Learning Health Systems, Korea University, Seoul, Republic of Korea

**Correction to: BMC Nurs (2022) 21:26**

**https://doi.org/10.1186/s12912-021-00803-z**

Following publication of the original article [1], the authors reported a typesetting error in Table 1, the content was mismatched in Article No. A8.

**Table 1:** Summary of the included studies

Article. No.	Author, year/Country	Research type	Aims	Sample size (F:M)	Age of participants (in years)	Nursing experience (years)	Working department	Data collection	Data analysis	Percentage that meets CASP
A1	Mealer et al., 2012/USA	Qualitative study	To identify mechanisms employed by highly resilient ICU nurses to develop preventative therapies to obviate the development of PTSD in ICU nurses	27, (27:0)	mean : 46	Total: mean18.5	Intensive Care Unit	Semi-structured telephone interviews	Thematic analysis	80%
A2	Shimoinaba et al., 2015/Japan	Qualitative study	To explore the nature of nurses’ resilience and the way it is developed	18, (18:0)	29-53 mean : 37.8	Total :7-26 In this department : 2-8, mean37.8	Palliative Care Unit	Face to face in-depth interviews	Grounded theory	80%
A3	Cope et al., 2016/Australia	Qualitative portraiture methodology	To explore residential aged care nurses working in interim, rehabilitation and residential aged care perceptions of resilience	3,(not reported),	32-57	Total :mean28	An aged care environment	Semi-structured interviews painting with words	Thematic analysis	70%
A4	Cope et al., 2016(2) /Australia	Qualitative portraiture methodology	To explore why nurses chose to remain in the Western Australian workforce; to develop insights into the role of resilience of nurses to manage the context of nursing work; and, to identify the key characteristics of resilience displayed by those nurses	9,(not reported)	Not reported	Not reported	Interim and residential aged care, academic setting, tertiary acute care setting	Individual interviews, field notes, memos and gesture drawings interviews	Phenomenology	70%
A5	Tubbert, 2016/USA	Qualitative study	To explore the resiliency characteristics of certified emergency nurses	16 (68.8%:31.2%)	Mean :50	Total :30 In this department : 20	Emergency department	Face to face interview	Content analysis	80%
A6	Benade et al., 2017/South Africa	Explorative descriptive qualitative research	To explore and describe the strengths and coping abilities of nurses caring for older persons and to formulate recommendations to strengthen their resilience	43 (43:0)	Not reported	Total : not reported In this department : <6month :2 1year<5years : 5 5years<10years : 4 >10years: 27	Aged care department in an urban environment	Focus group interview	Content analysis	70%
A7	Marie et al., 2017/UK	Interpretive qualitative design	To observe and describe the environment within community mental health workplaces, to explore the challenges facing Palestinian community mental health nurses (CMHNs) inside and outside their workplaces, and to examine their sources of resilience	15 (8:7)	24-60	Not reported	Mental health workplace	Face-to-face in depth interviews	Thematic analysis	80%
A8	Prosser et al., 2017/Canada	Interpretative phenomenological method	To understand how registered nurses in the acute psychiatric setting develop resilience to sustain his or her practice.	4(not reported)	Not reported	Total : 2-21 In this department : 2-16	Acute psychiatric units	Semi-structured face-to- face interview	Interpretative phenomenological analysis.	90%
A9	Wahab et al., 2017/Singapore	descriptive qualitative design using Photovoice	To explore the new graduate nurses’ accounts of resilience and the facilitating and impeding factors in building their resilience	9(6:3)	Mean : 24	Total: mean 1 In this department : not reported	Oncology, General Medicine, General Surgery, Psychiatry and Paediatric wards	Focus group interview, photographs	Content analysis	80%
A10	Imani et al., 2018/Iran	Phenomenology study	To explore Iranian hospital nurses’ lived experiences of intelligent resilience	10 (4:6)	34-52	Total : 11-28 In this department : not reported	Different types of wards	In-depth interview	The Colaizzi’s (1978) seven-step approach	70%
A11	Jackson et al., 2018/UK	Grounded theory	To better understand nurse burnout and resilience in response to workplace adversity in critical care	11(11:0),	20s:5, 30s:3, 40s:1, 50s:2	Total :4-36 In this department : not reported	Intensive care unit	Open-ended interviews	Grounded theory	90%
A12	Ramalisa et al., 2018/ South Africa	Empirical qualitative research	To explore and describe how to strengthen the resilience of nurses in a work environment with involuntary mental health care users.	24(not reported)	Not reported	Total :2-8 In this department : not reported	Psychiatric ward	Open-ended interview	Thematic analysis	80%
A13	Ang et al, 2019/Singapore	Qualitative grounded theory design	To generate a comprehensive account of the experiences of nurses as they cope with stress and demands of work, and to develop knowledge of the phenomenon of resilience among nurses.	15(15:3),	24-68 mean:38	Not reported	General hospital	Individual interviews	Glaserian constant comparison method	80%
A14	Ang et al., 2019(2)/ Singapore	Photovoice study	To explore the meaning of resilience to nurses and their perceived resilience enhancing factors	8(7:1)	27-68	Not reported	Accident and emergency department	Focus group interview , photo	Content analysis	80%
A15	Lin et al, 2019/Taiwan	Construction-grounded theory	To explore and understand the experiences of resilience among nurses in an overcrowded emergency department (ED)	13(13:0)	23-39	Total : not reported In this department : 2-17	Emergency department	In-depth interview	Construction-grounded theory	90%
A16	Udod et al, 2021/Canada	Qualitative study	To investigate the role stressors, and how coping strategies cultivated nurse managers’ resilience in rural workplaces.	16(15:1)	30s : 5 40s:9 Over 60:2	Total : mean4.6, 10-35 In this department : mean 7.28, 1-17	Rural site in western Canada	Individual semi-structured interview	Thematic analysis	80%

*F* female, *M* male, *CASP* Critical Appraisal Skills Programme checklist

The correct Table 1 has been provided in this Correction.

The original article [1] has been updated.
