# Supplementary material for: Exploring nurse perceptions and experiences of resilience: a meta-synthesis study
Source: BMC Nurs. 2022 Jan 19;21:26. doi: 10.1186/s12912-021-00803-z (PMC8766352; doi:10.1186/s12912-021-00803-z)
Supplement: Supplementary file 1 — Additional file 1. [file 12912_2021_803_MOESM1_ESM.docx]

Table S1. Search Strategy

| **Databases** | **Search No.** | **Query** | **Results** |
| --- | --- | --- | --- |
| **Pubmed (MeSH)** | #1 | (nurses[MeSH Terms]) OR (nurse*[Title/Abstract]) | 339,435 |
|  | #2 | (resilience, psychological[MeSH Terms]) OR (resilien*[Title/Abstract]) | 45,122 |
|  | #3 | (((((((qualitative research[MeSH Terms]) OR (Hermeneutics[MeSH Terms])) OR (Focus Groups[MeSH Terms])) OR (qualitative research[Title/Abstract])) OR (qualitative study[Title/Abstract])) OR (interview[Title/Abstract])) OR (experience[Title/Abstract])) OR (perception[Title/Abstract]) | 1,122,976 |
|  | #4 | (((nurses[MeSH Terms]) OR (nurse*[Title/Abstract])) AND ((resilience, psychological[MeSH Terms]) OR (resilien*[Title/Abstract]))) AND ((((((((qualitative research[MeSH Terms]) OR (Hermeneutics[MeSH Terms])) OR (Focus Groups[MeSH Terms])) OR (qualitative research[Title/Abstract])) OR (qualitative study[Title/Abstract])) OR (interview[Title/Abstract])) OR (experience[Title/Abstract])) OR (perception[Title/Abstract])) | 592 |
|  | #5 | #4 AND Filters applied: Humans, English, from 2011/1/1 - 2021/9/30 | **419** |
| **EMBASE (emtree)** | #1 | 'nurse'/exp OR ‘nurse’ | 404,076 |
|  | #2 | 'resilience'/exp OR 'resilience' | 43,125 |
|  | #3 | 'qualitative research'/exp OR 'qualitative research' | 106,156 |
|  | #4 | 'qualitative methods'/exp OR 'qualitative methods' | 9,759 |
|  | #5 | 'qualitative interview'/exp OR 'qualitative interview' | 4,131 |
|  | #6 | #3 OR #4 OR #5 | 115,031 |
|  | #7 | #1 AND #2 AND #6 | 200 |
|  | #5 | #4 AND [english]/lim AND [humans]/lim AND Publication date: from 2011 - 2021 | **188** |
| **CINAHL** | #1 | MM ( nurse or nurses or nursing ) OR TI ( nurse or nurses or nursing ) OR AB ( nurse or nurses or nursing ) | 598,902 |
|  | #2 | MM ( resilience or resiliency or resilient ) OR TI ( resilience or resiliency or resilient ) OR AB ( resilience or resiliency or resilient ) | 18,005 |
|  | #3 | MM ( qualitative research or qualitative study or qualitative methods or interview ) OR TI ( qualitative research or qualitative study or qualitative methods or interview ) OR AB ( qualitative research or qualitative study or qualitative methods or interview ) | 245,485 |
|  | #4 | #1 AND #2 AND #3 | 445 |
|  | #5 | #4 AND Limiters: publication date- from 2011/1/1 - 2021/9/30; English | **64** |
| **Web of Science** | #1 | ((TS=(nurse OR nurses OR nursing )) OR TI=(nurse OR nurses OR nursing )) OR AB=(nurse OR nurses OR nursing ) | 330,786 |
|  | #2 | ((TS=(resilience OR resilient OR resiliency)) OR TI=(resilience OR resilient OR resiliency)) OR AB=(resilience OR resilient OR resiliency) | 129,898 |
|  | #3 | ((TS=(qualitative research OR qualitative study OR qualitative method OR interview)) OR TI=(qualitative research OR qualitative study OR qualitative method OR interview)) OR AB=(qualitative research OR qualitative study OR qualitative method OR interview) | 874,067 |
|  | #4 | ((#1) AND #2) AND #3 | 715 |
|  | #5 | #4 AND Limiters: publication date- from 2011/1/1 - 2021/9/30; English | **347** |
| **PsycINFO** | #1 | TI ( nurse or nurses or nursing ) OR AB ( nurse or nurses or nursing ) OR KW ( nurse or nurses or nursing ) | 1,242 |
|  | #2 | TI ( resilience or resiliency or resilient ) OR AB ( resilience or resiliency or resilient ) OR KW ( resilience or resiliency or resilient ) | 1,688 |
|  | #3 | TI ( qualitative research or qualitative study or qualitative methods or interview ) OR AB ( qualitative research or qualitative study or qualitative methods or interview ) OR KW ( qualitative research or qualitative study or qualitative methods or interview ) | 8,885 |
|  | #4 | #1 AND #2 AND #3 | 2 |
|  | #5 | #4 AND Limiters: publication date- from 2011/1/1 - 2021/9/30; English | 2 |
